# Clinical validation of artificial intelligence-assisted karyotyping on peripheral blood in a cytogenetic diagnostic laboratory

**DOI:** 10.1007/s00439-025-02789-z

**Published:** 2025-11-10

**Authors:** Yujie Zhu, Matthew Hoi Kin Chau, Huilin Wang, Ning Song, Ran Wei, Kin Wah Suen, Anna Chi Sum Chan, Wan Ching Hung, Ye Cao, Zirui Dong, Tak Yeung Leung, Sau Wai Cheung, Kwong Wai Choy

**Affiliations:** 1 Department of Obstetrics & Gynaecology, The Chinese University of Hong Kong, Hong Kong SAR,, China; 2 Obstetrics and Gynecology Medical Center, Nanjing Drum Tower Hospital, Affiliated Hospital of Medical School,Nanjing University, Nanjing, China; 3 The Obstetric and Paediatric Center [Shenzhen] under Hong Kong Hub of Paediatric Excellence, The Chinese University of Hong Kong, Shenzhen,, China; 4 Shenzhen Research Institute, The Chinese University of Hong Kong, Shenzhen, China; 5 The Chinese University of Hong Kong-Baylor College of Medicine Joint Center for Medical Genetics, Hong Kong SAR,, China; 6 Maternal-Fetal Medicine Institute, Key Laboratory of Birth Defects Research, Birth Defects Prevention Research and Transformation Team, Bao’an Maternity and Child Health Hospital affiliated to Jinan University School of Medicine, Shenzhen, China; 7 Hangzhou Diagens Biotechnology Co., Ltd., Hangzhou, China; 8 Department of Obstetrics & Gynaecology, Prince of Wales Hospital, Hong Kong SAR,, China; 9 Fertility Preservation Research Center, Department of Obstetrics & Gynaecology, The Chinese University of Hong Kong, Hong Kong SAR,, China; 10 Department of Molecular and Human Genetics, Baylor College of Medicine, Houston, TX USA

## Abstract

G-banded chromosome analysis, also known as G-banded karyotyping, remains a fundamental and irreplaceable diagnostic modality in clinical genetic testing. G-banded karyotypes provide whole genome visualization through chromosome banding patterns at the single-cell resolution for the diagnosis of chromosomal disorders. However, this method is labor-intensive and requires specialized expertise to manually analyze and karyotype metaphase spreads. In recent years, artificial intelligence (AI) algorithms have been utilized to automate karyotyping and assist with chromosome analysis. Despite this progress, there is a scarcity of studies evaluating the utility of artificial intelligence-assisted (AI-assisted) karyotyping analysis in cytogenetics diagnostic laboratories. This study highlights promising applications of AI-assisted karyotyping analysis in a cytogenetics diagnostic laboratory through a combination of a literature review, our data, and experience from a retrospective cohort study. This study also discusses important considerations of the use of AI-assisted karyotyping analysis in a cytogenetic diagnostic laboratory and outlines a two-stage framework for its implementation into clinical workflows. This approach aims to utilize the accuracy and efficiency of AI-assisted karyotyping analysis, potentially benefiting personalized patient care and contributing to advancements in the health system.

**Supplementary Information:**

The online version contains supplementary material available at 10.1007/s00439-025-02789-z.

## Introduction

Chromosome analysis by G-banding has been a fundamental whole genome genetic diagnostic technique for over 50 years (Sumner et al. 1971; Natarajan 2002; Arora and Dhir 2016). Chromosomal disorders are a major category of genetic disease, accounting for a large proportion of all first-trimester pregnancy losses (47.4%) (Schilit et al. 2022), stillbirths (41.9%) (Reddy et al. 2012), children born with multiple congenital anomalies (39.3%) (El-Attar et al. 2021; Redin et al. 2017), and developmental delay. Moreover, chromosomal abnormalities are prevalent in couples with reproductive problems including infertility, recurrent miscarriage. Lastly, chromosome analysis is also considered a gold-standard method in diagnosing genetic aberrations in different cancers (Fröhling 2008). Chromosomal abnormalities can be categorized into numerical abnormalities (whole chromosome aneuploidies) and structural abnormalities (chromosome translocations, inversions, insertions, deletions, duplications, ring, and small supernumerary marker chromosomes (sSMC)). As such, G-banded chromosome analysis is performed for the genetic investigation of a wide range of clinical indications, providing crucial information for clinical diagnosis, prognosis clinical management, and reproductive risk.

Even with the advancements in molecular genomic technologies, such as chromosomal microarray (CMA), next-generation sequencing (NGS), and optical genome mapping (OGM), conventional chromosome analysis remains indispensable for the detection of balanced chromosome structural abnormalities (e.g. reciprocal and Robertsonian translocations, inversions), other chromosome structural abnormalities (e.g. isochromosomes, isodicentric chromosomes, ring chromosomes, and sSMC), complex chromosomal rearrangements, and mosaic abnormalities, which these newer techniques may not fully resolve (Chau et al. 2020). Despite its importance, karyotyping is a labor-intensive procedure that requires specialized expertise to analyze metaphase chromosomes by microscopy, followed by manual arrangement of chromosomes into karyograms for the detection of chromosomal abnormalities.

The advent of artificial intelligence has brought about new developments in healthcare across diverse disciplines. Deep Learning techniques have already been implemented in improving the accuracy of diagnostic medical imaging, particularly in radiology, pathology, genetic testing, and more (Kaur and Kaur 2022). For example, a multicenter study utilizing AI-assisted imaging enhanced diagnostic capabilities of thyroid cytopathologic diagnosis. AI-assisted image analysis increased the specificity of detection by cytopathologists from 88% to 99% and the accuracy improved from 87% to 94% (Wang et al. 2024). In an attempt to semi-automate karyotyping workflows, numerous computational classification modules have been developed for the analysis of metaphase chromosome images. Studies have showed evidence that Convolutional Neural Network (CNN) or Deep Neural Network (DNN) algorithms can aid karyotyping analysis (Jindal et al. 2017; Qin et al. 2019; Zhou et al. 2024a, b). More recently, AI has been incorporated into cytogenetic analysis software for automated chromosome counting, karyogram assembly, and abnormality detection. This presents an opportunity to improve efficiency and accuracy in clinical cytogenetics (Supplementary Table S1).

Potential applications of AI-assisted karyotyping analysis signify a new frontier in the field of cytogenetics. However, there is a lack of comprehensive studies assessing AI-assisted karyotyping in detecting constitutional chromosomal abnormalities in clinical practice. This study aims to explore the implementation of AI-assisted karyotyping analysis into clinical diagnostic workflows in a cytogenetics laboratory, including: (1) Clinical validation of the accuracy and efficiency of AI-assisted karyotyping in chromosome analysis; (2) Establishment of a two-stage framework to implement AI-assisted karyotyping in semiautomated cytogenetic analysis workflows, optimizing the accuracy and efficiency of it into clinical practice.

### Clinical validation of AI-assisted karyotyping analysis and considerations for clinical implementation

Following an exhaustive literature search on the use of AI in karyotyping in clinical applications (Supplementary Method 1), only two relevant articles were available. Both were single center studies from the mainland China. However, meta-analysis of the data was not performed because complete raw datasets were unavailable (Zhou et al. 2024a, b; Guo et al. 2024). The lack of robust validation data hinders the clinical implementation of AI-assisted karyotyping analysis. To bridge this gap, we performed a retrospective cohort study of AI-assisted karyotyping analysis in 100 peripheral blood samples, comprising of 50 abnormal and 50 normal cases for constitutional studies covering the period between January 2021 and December 2022 (Supplementary Table S2, Supplementary Figure S1). While successful karyotype analysis depends on multiple factors, including optimization of chromosome spread, banding quality, image resolution, this manuscript focuses on the application of AI-assisted karyotyping on blood cultures with approximately 550-banding level. This model development and validation was conducted by Diagens Biotechnology. The system utilizes three key AI algorithm modules: Cascade Mask R-CNN for chromosome segmentation and counting (Cai and Vasconcelos 2018), Varifocal-Net for chromosome classification and karyotype assembly (Qin et al. 2019), and HomNet for detection of chromosome structural abnormalities (Li et al. 2024). These models were developed based on deep learning techniques and have been trained and validated on large-scale clinical datasets. Additionally, we have explored key considerations to enhance its clinical application (Table 1). The detailed information with individual case-level and summary-level of the chromosome abnormalities are described in Supplementary Table S3 and S4.

**Table 1:** Key considerations for clinical application of AI-assisted karyotyping

Key consideration	Explanation
General applications of AI-assisted karyotyping to streamline cytogenetic workflows	1. Image selection and pre-processing:
	Automated evaluation and selection of high-quality grayscale images of metaphase spreads for downstream analyses
	Automated quality enhancement of metaphase images
	2. Automated metaphase counting, analysis and karyotype assembly:
	Accurately cuts chromosomes and segment overlapping or clustered chromosomes
	Accurately counts chromosomes
	Automated chromosome recognition and assembly of karyotypes
	These automated processes reduce hands-on analysis time for cytogeneticists, enhances efficiency, and boosts laboratory throughput, compared to traditional methods
Requirements for clinical implementation	1. Robust validations against conventional manual analysis among different sample types across various clinical applications are essential. This further establishes the accuracy, sensitivity, specificity, positive and negative predictive values of AI-assisted karyotyping performance
	2. Verify the sensitivity of AI-assisted karyotyping in the detection of a wide range of chromosomal abnormalities
	3. Quality control and external proficiency testing can identify areas of improvement across laboratories
	4. Standardized protocols are essential for consistent application of AI-assisted karyotyping for chromosome analysis across laboratories
Increase the sensitivity to detect low-level mosaic abnormalities	Automation via AI-assisted karyotyping enables a higher number of metaphases to be examined:
	Manual counting, analysis or scoring of 30 cells can exclude mosaicism of 10% at the 95% confidence level
	Automated metaphase counting, analysis, and karyotype assembly of 70 cells can exclude mosaicism of 5% at the 95% confidence level
Detection scope of chromosomal abnormalities	1. Numerical abnormalities: Yes, with an average accuracy of 99% (from literature review and our results)
	2. Structural abnormalities: Depends on AI-assisted karyotyping software, facing challenges and still requires improvements
Limitations and potential roles	1. Limited capability to distinguish cellular debris from suspected ring chromosome and sSMC
	2. Segmentation errors and misclassification of chromosomal abnormalities
	3. Limited capability in identifying subchromosomal structural abnormalities
	Chromosome analysis results are reported in accordance with the ISCN 2020 which requires experienced cytogeneticists, whose interpretation of these results is also crucial. Currently, karyograms assembled by AI-assisted karyotyping are not 100% accurate. An element of manual review for a subset of karyotypes must be implemented to the workflow

*sSMC* small supernumerary marker chromosome, *ISCN 2020* International System for Human Cytogenomic Nomenclature 2020

### AI-assisted karyotyping analysis assembles highly accurate karyotypes and boosts the efficiency of chromosome analysis workflows

The outcomes of our cohort study underscore the significant potential of AI-assisted karyotyping analysis for clinical implementation. AI-assisted karyotyping analysis performed automated selection of high-quality metaphase images, assembly of karyotypes and detection of chromosomal abnormalities. Comparing the results obtained by conventional analytical workflow, the results of AI-analysis without any manual corrections demonstrated an overall accuracy of 71%, with a sensitivity of 98%, specificity of 44%, positive predictive value (PPV) of 64%, and negative predictive value (NPV) of 96%. The specificity was lower because of misrecognition of cellular debris as chromosomes and identification of isolated metaphase spreads with random gains and losses of chromosomes, resulting in false positives. After conducting manual corrections, the corresponding values improved significantly. It achieved to an accuracy of 97%, sensitivity of 98%, specificity of 96%, PPV of 96%, and NPV of 98% (Table 2). The validation process within our cohort confirmed the consistency with high accuracy of AI-assisted karyotyping analysis with manual corrections versus conventional karyotyping, demonstrating capability to render metaphases images into individual chromosomes and assemble karyotypes correctly. AI-assisted karyotyping analysis also recognized chromosomal abnormalities including reciprocal translocations, Robertsonian translocations, and numerical abnormalities. However, the three cases flagged incorrectly by AI highlighted the need to acknowledge the current limitations of accuracy and reliability. Next, we compared the time required for the conventional analytical workflows versus AI-assisted karyotyping analysis (Supplementary Tables S1 ). The average time spent by each of the three cytogenetic technologists, with different years of practice, to count ten, analyze five, and karyotype two metaphases was around 33.9 ± 2.1 (mean ± SD) minutes. In contrast, the AI analysis was significantly faster, which clocked at 6.5 ± 0.5 (mean ± SD) minutes for counting, analyzing, and assembling karyotypes of 70 metaphases. This is the default parameter set by the software and we decided to keep this number to increase the number of metaphases to provide additional data points to assess the advantages and limitations of AI-assisted karyotyping on the metaphase level. Moreover, we assessed the possibility to detect low-level mosaic findings (Supplementary Table S5) (Hook 1977).

These findings demonstrate that AI-analysis significantly shortened hands-on analysis time compared with conventional manual analysis (*P* < 0.001). The time spent by cytogenetic technologists to manually review 15 representative karyotypes after AI analysis was 7.0 ± 1.0 (mean ± SD) minutes. These results unveiled remarkable gains in time efficiency for chromosome analysis through the utilization of AI-assisted karyotyping analysis, ultimately leading to a shorter turnaround time (TAT).

Although a low number of aneuploid metaphases were detected by AI-assisted karyotyping analysis in seven cases, none reached significant reportable levels. These findings are likely attributable to the increased metaphases analyzed by AI and their clinical significance remains uncertain (Supplementary Table S6).

**Table 2:** Accuracy, sensitivity, specificity, positive and negative predictive values of AI analysis before and after manual corrections

Performance of AI analysis	Accuracy (%)	Sensitivity (%)	Specificity (%)	PPV (%)	NPV (%)
AI analysis before manual corrections*	71%	98%	44%	64%	96%
AI analysis after manual corrections*	97%	98%	96%	96%	98%

*PPV* positive predictive value, *NPV* negative predictive value

* Manual corrections were defined as correction of misrecognition of cellular debris as chromosomes and removal of isolated metaphase spreads with random gains and losses of chromosomes, reducing false positive calls from the AI karyotyping software

### Challenges for the clinical implementation of AI-assisted karyotyping analysis

We present unique challenges in the current practice after integration of AI-assisted karyotyping analysis: First, it is challenging for AI-assisted karyotyping analysis to distinguish cellular debris from sSMC or ring chromosomes. During culturing, harvesting and slide preparation, cellular debris and interphase cells can accumulate in the surrounding environment. These debris comprise residues from cytoplasmic, interphase nuclei or damaged cells, as well as fragmented chromosomes, staining debris, and other organic or inorganic impurities. It is important to note that certain debris can be challenging to distinguish by AI-assisted karyotyping analysis due to their resemblance to stained chromatin material. Over-filtering may lead to loss of important information and under filtering may lead to inclusion of debris into karyotypes.

The second challenge is the incorrect segmentation and misclassification of chromosomes particularly in overlapping chromosome clusters or chromosomes that fold or twist (Arora and Dhir 2016; Kaur and Dhir 2023). Even chromosomes from the same preparation can vary in banding resolution, shapes, sizes, and orientations, posing challenges for accurate classification. These factors confound the ability of the software to precisely segment and classify chromosomes in difficult clusters.

The third challenge is the limited capability of AI-assisted karyotyping analysis in identifying subchromosomal abnormalities and intricate structural abnormalities. This limitation primarily arises from the diverse and unique abnormal morphologies of chromosome abnormalities. The second reason may be insufficient training dataset representative of a broad range of structural abnormalities in the model development stage. Our findings indicate that AI-assisted karyotyping analysis currently lacks the capability to flag certain Robertsonian translocations like 45,XX,der(22;22)(q10;q10) and 45,XX,der(15;21)(q10;q10).

### Streamlining and changing conventional clinical workflow with AI-assisted karyotyping analysis

**Fig. 1:**
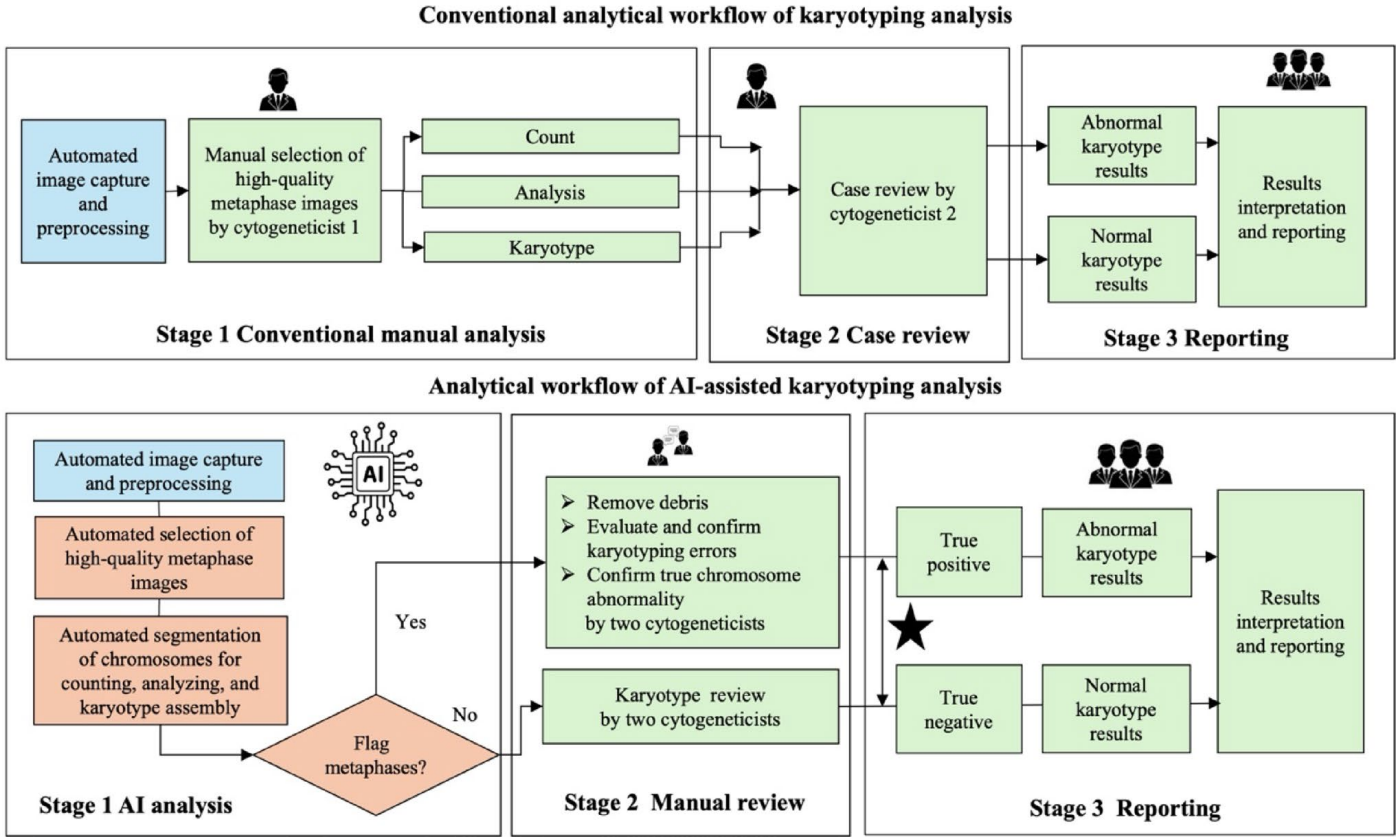
Augmented clinical workflow with AI-assisted karyotyping in chromosome analysis. The blue boxes focus on the computer-aided systems, such as image selection and pre-processing. Red boxes highlight AI-assisted karyotyping analysis processes through automated metaphase counting, analysis and karyotyping. Green boxes indicate manual work by cytogenetic technologists and directors. ★ AI-assisted karyotyping analysis currently flags some metaphases incorrectly, leading to false positive. Additionally, it lacks the capability to flag certain structural abnormalities, resulting in false negative results. *AI* artificial intelligence

To understand how AI-assisted karyotyping analysis might improve cytogeneticists’ experience at a tertiary prenatal diagnosis center, we begin by reviewing the conventional workflow and to further outline new clinical workflow (Fig. 1). The AI-assisted karyotyping analysis consists of two stages: stage one accurately identifies and flags problematic karyotypes, directing cytogeneticists’ attention to potentially abnormal cases and thereby streamlining the review process. The manual review, designated as stage two, is further categorized into three distinct types:

### Remove debris

**Fig. 2:**
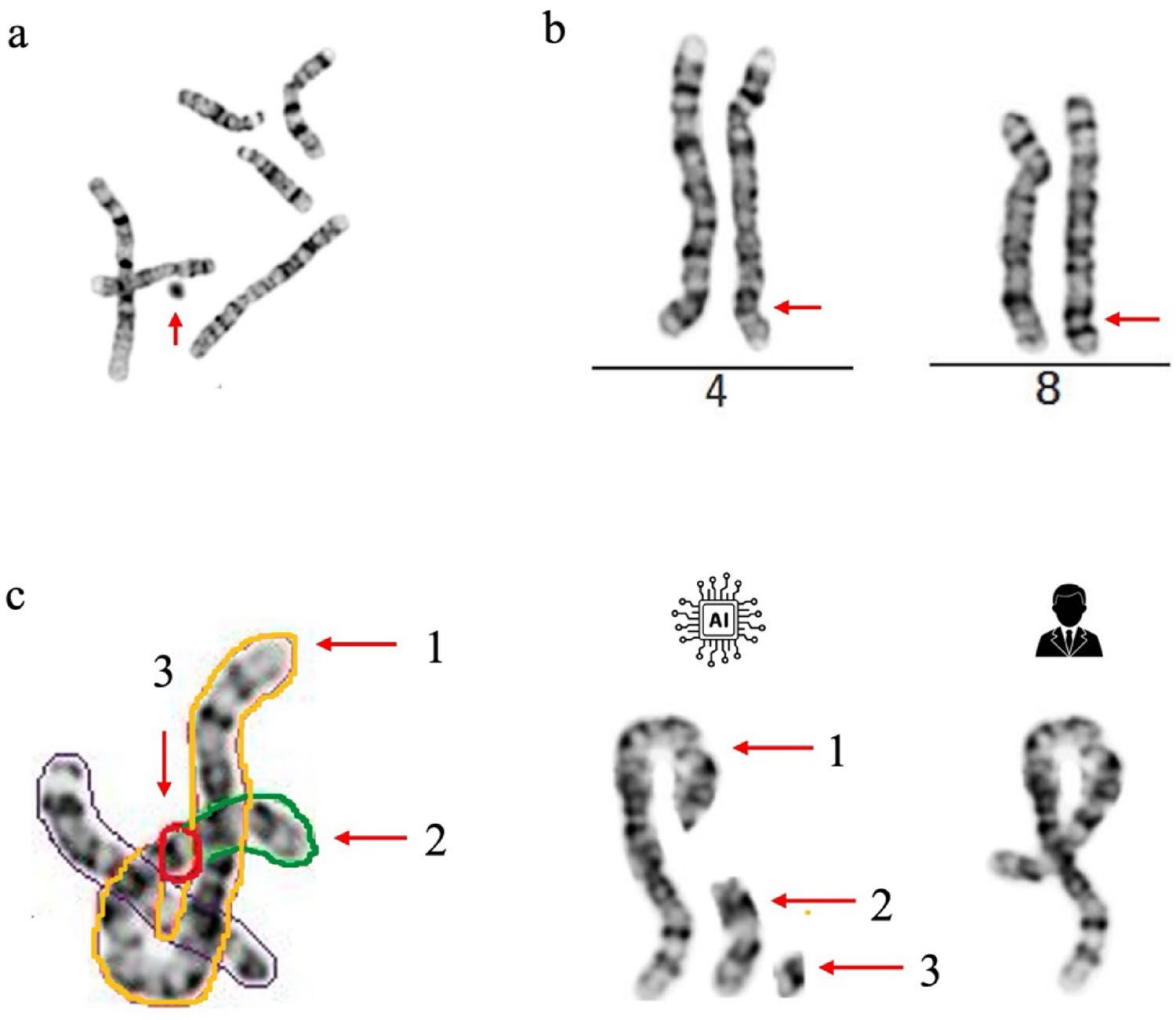
Examples on debris and karyotyping assembly errors. **a** Debris: The red arrow indicates the presence of debris misclassified as a chromosome by AI-assisted karyotyping analysis. **b** Reciprocal translocation of 46,XX,t(4;8)(q33;q24.1) was undetected by AI-assisted karyotyping analysis: the red arrow indicates the breakpoints. **c** Incorrect segmentation of twisted chromosomes: AI-assisted karyotyping analysis segmented a twisted chromosome 2 into three separate chromosomes, the red arrow indicates the segmentations

Debris, appreciable as dark-gray or black debris in metaphase images may be incorrectly recognized as sSMC by the AI-assisted karyotyping analysis (Fig. 2a). Hence, in the clinical practice, regular monitoring of cell cultures with media changes and culture rinsing is needed to eliminate blood and cellular debris. During cell harvest, hypotonic solution and fixation facilitates optimal chromosome spread, thereby improving banding quality and minimize debris. In the realm of image optimization, preprocessing steps play a vital role in filtering out debris to ensure accurate chromosome recognition and segmentation. The most used techniques are noise removal and contrast enhancement in AI-assisted karyotyping analysis. Cytotechnologist meticulously navigate through step one, managing and filtering these debris, thereby removing background noise and objects, while recognizing true sSMC and ring chromosomes. Further confirmation of sSMCs and rings can be supported with FISH (Mascarello et al. 2011) and Chromosomal Microarray Analysis (CMA) (Miller et al. 2010).

### Evaluate and confirm karyotyping errors

The AI-assisted karyotyping analysis may produce errors in karyotyping assembly, including: (1) Failure to detect chromosomal abnormalities (Fig. 2b); (2) Incorrect segmentation of twisted and overlapping chromosome clusters (Fig. 2c).

Despite the implementation of DNN for chromosome segmentation (Saleh et al. 2019; Liu et al. 2024), precise feature extraction remains a challenge. Previous studies have revealed that some intelligent assistant systems aim to detect and classify a wide range of structural abnormalities through homologous similarity (Supplementary Figure S2). However, the sensitivity is not optimal (Li et al. 2024), detecting chromosomal structural abnormalities such as subchromosomal deletions, duplications, inversions, and chromosomal translocations remain challenging. Therefore, an element of manual review by cytogeneticists must be implemented in the AI-assisted karyotyping analysis workflow to re-evaluate, re-segment, and to recognize chromosomal abnormalities that were undetected by AI-assisted karyotyping analyses at the present time.

### Confirmation of true chromosome abnormality

AI-assisted karyotyping analysis flags potentially abnormal metaphases including numerical and structural abnormality. Following a review of the karyotypes, cytogeneticists can judge whether the flagged case/metaphases contain a true chromosomal abnormality. The integration of other laboratory results, such as CMA, Low-pass Genome Sequencing (Low-pass GS) (Chau et al. 2022; Dong et al. 2017, 2019), and Optical Genome Mapping (OGM) (Mantere et al. 2021) are expected to delve deeper insights and develop a comprehensive diagnostic result when specific laboratory findings or clinical indications are present. The new clinical workflow provides clearer guidance on how cytogeneticists can leverage AI analysis to identify and diagnose abnormal cases more efficiently, while maintaining the necessary levels of manual review. On average, three ± two flagged metaphases by AI-assisted karyotyping analysis needs to be resolved in blood karyotyped images for each case. After AI analysis with manual review, the accuracy was 100% concordant with the results of conventional analysis.

## Conclusion

This work provides evidence for the application of AI-assisted karyotyping analysis as a semiautomated workflow in a cytogenetics diagnostic laboratory. AI-assisted karyotyping analysis demonstrates high accuracy with the technology’s potential to improve productivity, shorten TAT, increase the number of cells analyzed, and enable faster intervention. While time efficiency is important in cytogenetic diagnostics, accuracy and reproducibility are paramount for patient care.AI cannot currently replace cytogenetic technologists, the results prioritization and optimization of AI-assisted karyotyping analysis allow cytogeneticists to focus their expertise to more complex anomalies during manual review. 

These benefits may lead to cutting down of costs, facilitating the benefit of karyotyping analysis to a broader population, promoting health equity across diverse populations and ultimately reducing the burden on the entire healthcare system. The market for karyotyping services is expected to maintain a fundamental and irreplaceable position in clinical genetic testing, and the Asia-Pacific region is anticipated to exhibit the fastest growth during the period of 2023 to 2032. AI-assisted karyotyping analysis is poised to meet the growing demand in the healthcare system by enhancing laboratory throughput via optimizing speed, efficiency, cost-effectiveness, and resource optimization.

### Limitation

This is a preliminary study with several notable limitations. First, the cohort comprises a relatively small sample of 100 cases from a single hospital. Additionally, the validation focused solely on chromosome analyses of peripheral blood specimens from adult patients undergoing investigation for reproductive abnormalities. The data predominantly addresses relatively simple chromosomal abnormalities. Further investigation in large-scale studies among various sample types and assessing the ability of AI-assisted karyotyping to detect a broader spectrum of chromosomal abnormalities are required to facilitate implementation across different clinical applications.

## Supplementary Information

Below is the link to the electronic supplementary material.

Supplementary Material 1

Supplementary Material 2

## Data Availability

No datasets were generated or analysed during the current study.

## References

[CR3] ACMG (2024) Technical standards for clinical genetics laboratories section E: clinical cytogenetics. https://www.acmg.net/PDFLibrary/Updated%20Section%20E%202024.pdf. Accessed 26 May 2025

[CR1] Arora T, Dhir R (2016) A review of metaphase chromosome image selection techniques for automatic karyotype generation. Med Biol Eng Comput 54:1147–1157. 10.1007/s11517-015-1419-z 26676686 10.1007/s11517-015-1419-z

[CR4] Cai Z, Vasconcelos N (2018) Cascade R-CNN: delving into high quality object detection. In: IEEE/CVFconference on computer vision and pattern recognition, Salt Lake City, UT, USA, 2018, pp.6154-6162. 10.1109/CVPR.2018.00644

[CR6] Chau MHK, Wang H, Lai Y et al (2020) Low-pass genome sequencing: a validated method in clinical cytogenetics. Hum Genet 139:1403–1415. 10.1007/s00439-020-02185-9 32451733 10.1007/s00439-020-02185-9

[CR7] Chau MHK, Li Y, Dai P et al (2022) Investigation of the genetic etiology in male infertility with apparently balanced chromosomal structural rearrangements by genome sequencing. Asian J Androl 24:248–254. 10.4103/aja2021106 35017386 10.4103/aja2021106 PMC9226698

[CR9] Dong Z, Xie W, Chen H et al (2017) Copy-number variants detection by low‐pass whole‐genome sequencing. Curr Protoc Hum Genet 94(1):8–17. 10.1002/cphg.43 10.1002/cphg.43 28696555

[CR10] Dong Z, Zhao X, Li Q et al (2019) Development of coupling controlled polymerizations by adapter-ligation in mate-pair sequencing for detection of various genomic variants in one single assay. DNA Res 26:313–325. 10.1093/dnares/dsz011 31173071 10.1093/dnares/dsz011 PMC6704401

[CR11] El-Attar LM, Bahashwan AA, Bakhsh AD et al (2021) The prevalence and patterns of. chromosome abnormalities in newborns with major congenital anomalies: a retrospective study from Saudi Arabia. Intractable Rare Dis Res 10:81–87. 10.5582/irdr.2021.01016 33996352 10.5582/irdr.2021.01016 PMC8122309

[CR12] Fröhling S, Döhner H (2008) Chromosomal abnormalities in cancer. N Engl J Med 359:722–734. 10.1056/NEJMra0803109 18703475 10.1056/NEJMra0803109

[CR5] Guo C, Wang J, Yang L et al (2024) Application of artificial Intelligence-assisted chromosome karyotyping analysis in prenatal diagnosis. Chin Gen Pract 27:2883–2887. 10.12114/j.issn.1007-9572.2023.0549

[CR14] Hook EB (1977) Exclusion of chromosomal mosaicism: tables of 90%, 95% and 99% confidence limits and comments on use. Am J Hum Genet 29:94–97 835578 PMC1685228

[CR29] Jindal S, Gupta G, Yadav M et al (2017) Siamese networks for chromosome classification. In: Proceedings of the IEEE international conference on computer vision workshops (ICCV), pp 72–81. 10.1109/ICCVW.2017.17

[CR16] Kaur K, Dhir R (2023) A study of machine learning techniques for automated karyotyping system. BioRxiv. 10.1101/2023.11.16.56747337961145

[CR15] Kaur J, Kaur P (2022) Outbreak COVID-19 in medical image processing using deep learning: a state-of-the-art review. Arch Comput Methods Eng 29:2351–2382. 10.1007/s11831-021-09667-7 34690493 10.1007/s11831-021-09667-7 PMC8525064

[CR17] Li J, Fu F, Wei R et al (2024) Chromosomal structural abnormality diagnosis by homologous similarity. In: Proceedings of the 30th ACM SIGKDD conference on knowledge discovery and data mining, pp 5317–5328. 10.1145/3637528.3671642

[CR18] Liu X, Wang S, Lin JC-W, Liu S (2024) An algorithm for overlapping chromosome segmentation based on region selection. Neural Comput Appl 36:133–142. 10.1007/s00521-022-07317-y

[CR19] Mantere T, Neveling K, Pebrel-Richard C et al (2021) Optical genome mapping enables constitutional chromosomal aberration detection. Am J Hum Genet 108:1409–1422. 10.1016/j.ajhg.2021.05.012 34237280 10.1016/j.ajhg.2021.05.012 PMC8387289

[CR21] Mascarello JT, Hirsch B, Kearney HM et al (2011) Section E9 of the American college of medical genetics technical standards and guidelines: fluorescence in situ hybridization. Genet Med 13:667–675. 10.1097/GIM.0b013e3182227295 21738013 10.1097/GIM.0b013e3182227295

[CR22] Miller DT, Adam MP, Aradhya S et al (2010) Consensus statement: chromosomal microarray is a First-Tier clinical diagnostic test for individuals with developmental disabilities or congenital anomalies. Am J Hum Genet 86:749–764. 10.1016/j.ajhg.2010.04.006 20466091 10.1016/j.ajhg.2010.04.006 PMC2869000

[CR23] Natarajan AT (2002) Chromosome aberrations: past, present and future. Mutat Res 504:3–16. 10.1016/S0027-5107(02)00075-1 12106642 10.1016/s0027-5107(02)00075-1

[CR24] Qin Y, Wen J, Zheng H et al (2019) Varifocal-Net: A chromosome classification approach using deep convolutional networks. IEEE Trans Med Imaging 38:2569–2581. 10.1109/TMI.2019.2905841 30908259 10.1109/TMI.2019.2905841

[CR25] Reddy UM, Page GP, Saade GR et al (2012) Karyotype versus microarray testing for genetic abnormalities after stillbirth. N Engl J Med 367:2185–2193. 10.1056/NEJMoa1201569 23215556 10.1056/NEJMoa1201569 PMC4295117

[CR26] Redin C, Brand H, Collins RL et al (2017) The genomic landscape of balanced cytogenetic abnormalities associated with human congenital anomalies. Nat Genet 49:36–45. 10.1038/ng.3720 27841880 10.1038/ng.3720 PMC5307971

[CR30] Saleh HM, Saad NH, Isa NAM (2019) Overlapping chromosome segmentation using U-Net: convolutional networks with test time augmentation. Procedia Comput Sci 159:524–533. 10.1016/j.procs.2019.09.207

[CR31] Schilit SLP, Studwell C, Flatley P et al (2022) Chromosomal microarray analysis in pregnancy loss: is it time for a consensus approach? Prenat Diagn 42:1545–1553. 10.1002/pd.6244 36176068 10.1002/pd.6244

[CR28] Sumner AT, Evans HJ, Buckland RA (1971) New technique for distinguishing between human chromosomes. Nat New Biol 232:31–32. 10.1038/newbio232031a0 4105244 10.1038/newbio232031a0

[CR32] Wang J, Zheng N, Wan H et al (2024) Deep learning models for thyroid nodules diagnosis of fine-needle aspiration biopsy: a retrospective, prospective, multicentre study in China. Lancet Digit Health 6:e458–e469. 10.1016/S2589-7500(24)00085-2 38849291 10.1016/S2589-7500(24)00085-2

[CR33] Zhou C, Wang J, Xiang S et al (2024a) A simple normalization technique using window statistics to improve the Out-of-Distribution generalization on medical images. IEEE Trans Med Imaging 43:2086–2097. 10.1109/TMI.2024.3353800 38224511 10.1109/TMI.2024.3353800

[CR34] Zhou Y, Xu L, Zhang L et al (2024b) Enhancing chromosomal analysis efficiency through deep learning-based artificial intelligence graphic analysis. Discov Appl Sci 6:299. 10.1007/s42452-024-05980-5

