# Supplementary material for: Clinical validation of artificial intelligence-assisted karyotyping on peripheral blood in a cytogenetic diagnostic laboratory
Source: Hum Genet. 2025 Nov 10;144(11-12):1269–76. doi: 10.1007/s00439-025-02789-z (PMC12689769; doi:10.1007/s00439-025-02789-z)
Supplement: Supplementary file 1 — Supplementary Material 1 [file 439_2025_2789_MOESM1_ESM.pdf]

Supplementary information

Clinical validation of artificial intelligence-assisted karyotyping on peripheral blood in a cytogenetic diagnostic laboratory

Yujie Zhu[1,2,3]* • Matthew Hoi Kin Chau[1,3,4,5]* • Huilin Wang[3,4,6] • Ning Song[7] • Ran Wei[7] • Kin Wah Suen[1] • Anna Chi Sum Chan[8] • Wan Ching Hung[1] • Ye Cao[1,3,4] • Zirui Dong[1,3,4,9] • Tak Yeung Leung[1,4,5] • Sau Wai Cheung[5,10]† • Kwong Wai Choy[1,3,4,5,9] †

***Joint first authors; †Joint last authors**

Correspondence Author:

Richard Kwong Wai Choy, PhD, Women and Children's Medical Center (Shenzhen), Hong Kong Hub of Paediatric Excellence, Department of Obstetrics & Gynaecology, The Chinese University of Hong Kong, Hong Kong SAR, China

Tel: +852 3505 3099

Fax: +852 2636 0008

richardchoy@cuhk.edu.hk

Supplementary Method 1

Information on the search strategy and the selection criteria

We conducted a comprehensive review in June 2024 by searching the MEDLINE database via PubMed for articles published between Jan 2000, and June 2024, with the following search terms: (“Karyotype analysis” [all fields]) AND (“Artificial intelligence” [all fields]). Additionally, we utilized Google to identify relevant articles published between Jan 2000, and June 2024 using the keyword “AI” and “karyotyping. Furthermore, we searched on Google Scholar database in June 2024, using the search terms related to ("Karyotype analysis" OR "Chromosome analysis" OR "G-banding") AND ("Machine learning" OR "Artificial intelligence" OR "AI" OR "Neural network" OR "Deep neural network" OR "Recurrent neural network" OR "Convolutional neural network") AND ("Framework" OR "Clinical practice" OR "Regulation") in all fields setting. Our search was extended to the Web of Science database in June 2024, with the key words “Karyotype analysis” and “Artificial Intelligence” published between Jan 2000 and June 2024. Subsequently, the search was re-runned and updated with the same parameters in July 2024. To complement our database searches, we manually screened the references of pertinent articles.

In conducting our scoping literature review, we systematically searched for primary research, opinion articles, abstracts and reviews published from database inception. No language restrictions were used in any of our searches. The eligibility criteria for the studies we selected focused on the articles in evaluating and validating the clinical accuracy and effectiveness of AI-based systems utilizing CNN or DNN algorithms in the chromosome karyotyping analysis, in collaboration with hospitals and institutions.

Supplemental Tables

Table S1. Definitions and specifications of AI-assisted karyotyping used in our study

Category	Information
Currently available AI-assisted karyotyping software	1. HiBand scanning and analysis system (Applied Spectral Imaging, CA, USA)<br>2. Ikaros karyotyping platform (MetaSystems, Altlussheim, Germany)<br>3. AutoVision® Intelligent Chromosome Analysis System (Diagens, Hangzhou, China)<br>4. CytoVision® (Genetix Leica Microsystems, New Milton, England)<br>5. ArgusSoft karyotyping software (ArgusSoft, Saint Petersburg, Russia)<br>6. MetaClass karyotyping (Microptic, Barcelona, Spain)
AI-assisted karyotyping software used in our study	1. Conventional karyotyping analysis: Leica GSL-120 System (Leica Biosystems, Nussloch, Germany)<br>2. AI-assisted karyotyping analysis: Auto Vision® Intelligent Chromosome Analysis System (Version: 2.0.6.2, Hangzhou Diagens Biotechnology Co., Ltd. Mainland China)
Detailed specifications of AI models from AI-assisted karyotyping used in our study	1. Model for chromosome classification: Varifocal-Net (Qin et al.2019)<br>Training datasets: 1909 cases from Xiangya Hospital, encompassing normal and abnormal samples<br>Mechanism: It blends global and local chromosome traits using a convolutional neural network split into G-Net and L-Net for simultaneous classification of chromosomes type and polarity<br>Internal evaluation: 99.2% accuracy<br>2. Model for chromosomal structural abnormality: HomNet (Li et al. 2024)<br>Mechanism: It aligns homologous chromosomes and identifies structural abnormalities through homologous similarity<br>External evaluation: It showcased a 75% sensitivity, a 99% specificity, and a 98.4% diagnostic accuracy
Definition of conventional analytical workflow in our study	According to National Pathology Accreditation Advisory Council (Third Edition 2013) "Requirements for Cytogenetic Testing"; Commonwealth of Australia, routine karyotyping manual analysis * standards are as follows:<br>1. Count: A minimum of 15 metaphases counted<br>2. Analyze: At least 5 banded cells analyzed<br>3. Karyotype: 2 of the fully analyzed metaphases with karyograms<br>All karyotypes are reported according to the ISCN 2020, and all images are archived as part of the patient's laboratory record to allow retrieval of sufficient information to confirm the result

	*Manual analysis served as the gold reference standard for evaluating the AI-assisted karyotyping workflow
AI-assisted karyotyping flags potentially abnormal metaphases in our study	The software performed automated counts, analyses, and karyotype assemblies of 70 cells, simultaneously flagging potentially abnormal metaphases that contain numerical and/or structural abnormalities. This assists cytogeneticists in the effective identification of abnormal cases.
Definition of positive and negative calls by AI-assisted karyotyping in our study	1. Positive call: The case contains flagged metaphases by AI analysis, indicating potential abnormalities<br>2. Negative call: The case does not contain flagged metaphases by AI analysis, indicating absence of abnormalities
Definition of manual review in our study	Manual review includes the steps in manual correction (correct the misrecognition of cellular debris as chromosomes and removal of isolated metaphase spreads with random gains and losses of chromosomes) and then evaluate and confirm karyotyping errors
The differences in accuracy and objectives between the AI analysis after manual corrections and manual review	1. The accuracy of AI analysis after manual corrections is 97%, aiming to address the limitation for accuracy and reliability.<br>2. The accuracy of AI analysis after manual review is 100%, aiming to ensure its implementation into clinical practice.
Clinical evaluation metrics of AI-assisted karyotyping in our study	We first define the following four criteria:<br>1. TP: Cases flagged with positive call by AI analysis which is consistent with the gold standard<br>2. TN: Cases flagged with negative call by AI analysis which is consistent with the gold standard<br>3. FP: Cases flagged with positive call by AI analysis which is inconsistent with the gold standard<br>4. FN: Cases flagged with negative call by AI analysis which is inconsistent with the gold standard

NOTE: TP = True positive. TN = True negative. FP = False positive. FN = False negative.

ISCN 2020 = International System for Human cytogenomic Nomenclature 2020.

Table S2. Detailed clinical indications of validation dataset

	Detailed clinical indications	Normal	Abnormal	Total
1	Recurrent abortion or RM	18	6	24
2	Translocation carrier or history of adverse pregnancy outcome or indications for fetal anomalies	4	19	23
3	Recurrent abortion or RM with AMA	6	9	15
4	Infertility (Severe oligospermia/Primary ovarian insufficiency)	6	4	10
5	RIF	6	1	7
6	AMA only	3	4	7
7	NIPT positive	3	5	8
8	Work up for IVF or PGT	4	2	6

NOTE: IVF = In vitro fertilization. RIF = Repeated implantation failure. AMA = Advanced maternal age. PGT = Preimplantation genetic testing. RM = Recurrent miscarriage. NIPT = Non-invasive prenatal testing.

Table S4. Summary-level data of the chromosomal abnormalities

Types of Karyotype Images (100 cases)			Sample Type	Chromosome Banding Resolution
Normal Karyotype Cases (50 cases: 27 Male and 23 Female)			Peripheral Blood	550 banding
Abnormal Karyotype Cases (50 cases:17 male and 33 Female)	Numerical Abnormalities (2 cases)	Sex Chromosome Abnormality (2 cases)	Peripheral Blood	550 banding
	Structural Abnormalities (45 cases)	Balanced Translocation (13 cases)	Peripheral Blood	550 banding
		Inversion 9 (12 cases)	Peripheral Blood	550 banding
		Robertsonian Translocation (20cases)	Peripheral Blood	550 banding
	Mosaicism	Low-level Mosaicism (3 cases)	Peripheral Blood	550 banding

Table S5. Percentage of mosaicism excluded with 90%, 95%, and 99% confidence levels, specified by the number of cells evaluated and found to have identical karyotypes

No. Cells (n)	Confidence Levels		
	90%	95%	99%
15	15%	19%	27%
64-73	4%	5%	7%

NOTE: When mosaicism is suspected, conventional manual analysis of 30 cells is typically examined during the karyotyping process. However, even with this more extensive analysis, our ability to detect mosaicism remains constrained. With the assistance of AI-Assisted karyotyping, mosaicism affecting 5% or more of the cells can be ruled out with 95% confidence by counting 64-73 cells as reported by Hook and colleagues (Hook 1977).

Table S6. Low numbers of abnormal metaphases detected by AI analysis and confirmation by manual review and microscope

Clinical Indication	Age	Conventional Karyotype result	# of abnormal metaphases by AI analysis	# of abnormal metaphases confirmed by manual review and microscope	Reason for discrepancy
RIF	42	46,XY	2 counts of 47,XYY,<br>2 counts of 45,X	1 count of 45,X	• Debris misclassified as chromosome Y in two cells<br>• Out-of-frame chromosome Y in one cell
RA	33	46,XY	2 counts of 47,XYY,<br>2 counts of 45,X	1 count of 45,X	• Debris misclassified as chromosome Y in two cells<br>• Out-of-frame chromosome Y in one

					cell
RIF (wife)	43	46,XY	3 counts of 47,XYY,<br>1 count of 47,XXY	1 count of 47,XXY	• Debris misclassified as chromosome Y in three cells
IVF and PGT-A	50	46,XY	2 counts of 47,XYY,<br>2 counts of 45,X	2 counts of 45,X	• Debris misclassified as chromosome Y in one cell<br>• Segmentation error misclassified as chromosome Y in one cell
Miscarriage	35	46,XX	2 counts of 45,X	2 counts of 45,X	• Consistent
RIF	36	46,XX	2 counts of 47,XXY,<br>2 counts of 45,X	1 count of 45,X	• Debris misclassified as chromosome Y in two cells<br>• Out-of-frame chromosome X in one cell
AMA	40	46,XX	2 counts of 45,X	1 count of 45,X	• Out-of-frame chromosome X in one cell

# = numbers. M = Male. F = Female. RIF = Repeated implantation failure. RA=Recurrent abortion. IVF = In vitro fertilization. PGT-A = Preimplantation genetic testing for aneuploidy. AMA = Advanced maternal age.

Supplemental Figures

Figure S1. Workflow of our cohort study

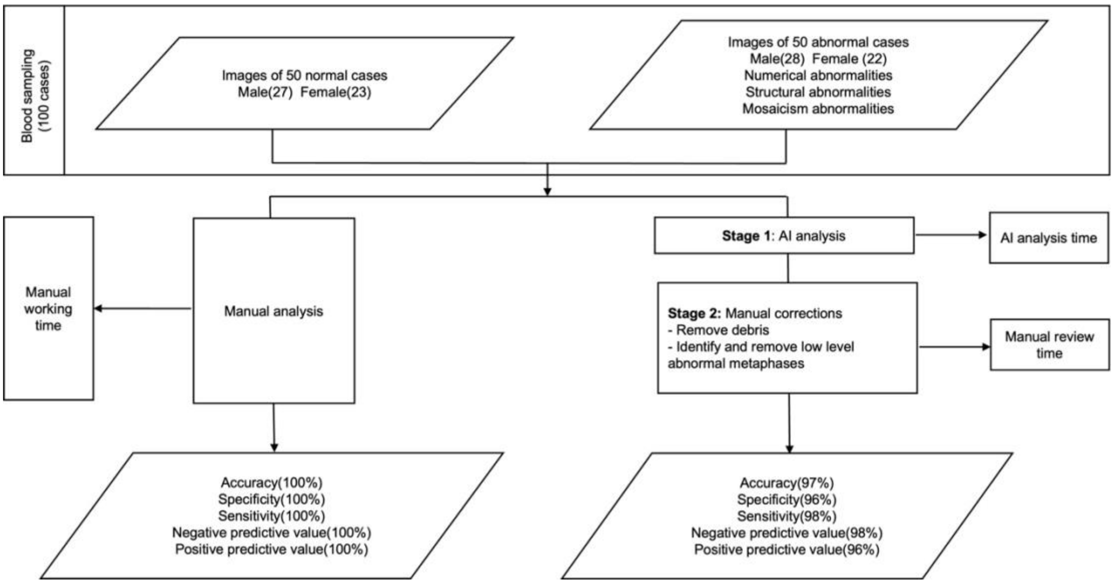

This workflow showed that a total of 100 cases were included in the validation cohort, comprising 50 abnormal and 50 normal cases. The diagnostic accuracy with sensitivity, specificity, negative predictive value (NPV) and positive predictive value (PPV) of AI-analysis before and after manual corrections was compared separately with the results obtained by conventional analytical workflow. Additionally, we compared the time required to count, analyze, and assemble karyotype manually versus AI-Assisted karyotyping. We further illustrated it significantly improve detection rate of low-level mosaicism and clarified the low numbers of abnormal metaphases.

Figure S2. Detailed examples of positive and negative call with AI analysis

(a) Positive call with AI analysis

Conventional manual analysis: 47,XXX

Positive call in numerical abnormality: AI-assisted karyotyping provided a detailed comment stating: "Analyzed 70 cells, among which 37 cells have suspected abnormal number of Sex Chromosomes. Please pay special attention," while flagging potential numerical abnormality in each metaphase image.

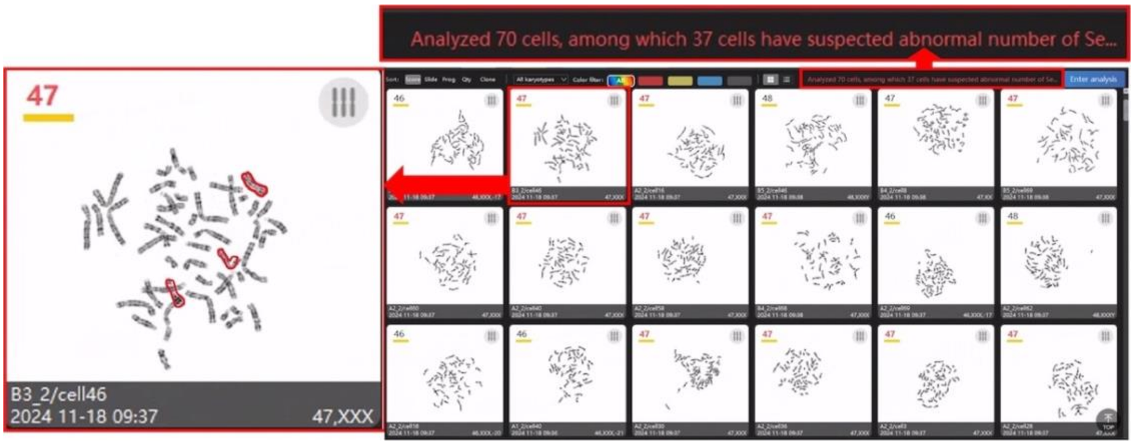

Conventional manual analysis: 46,XX,t(4;14)(q27;q32.3)

Positive call in structural abnormality: AI-assisted karyotyping provided a detailed comment stating: "Analyzed 70 cells, among which 56 cells are suspected to have structural abnormalities in chromosome 4 and chromosome 14. Please pay special attention", while flagging potential structural abnormality in each metaphase image.

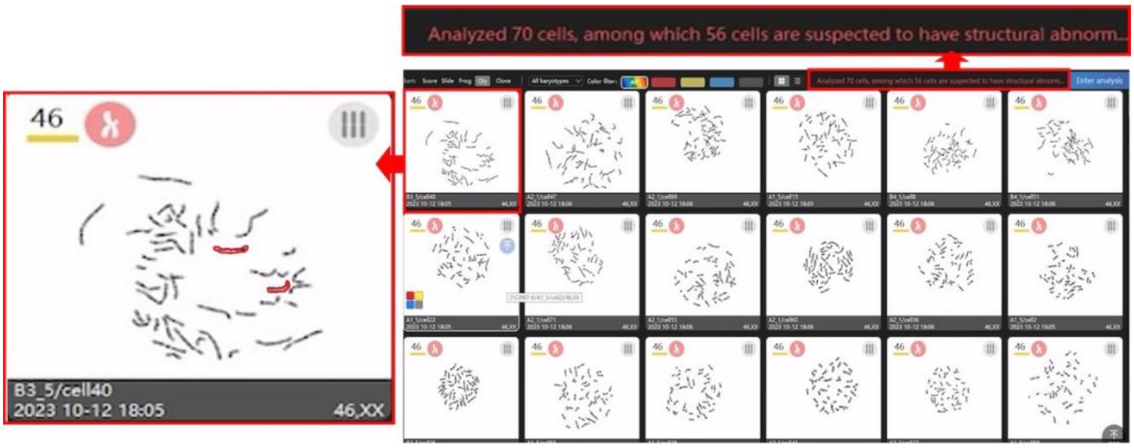

(b) Negative call with AI analysis

Conventional manual analysis: 46,XX

Negative call in normal case: AI-assisted karyotyping provided no comments on the potential normal case, without flagging any metaphase images.

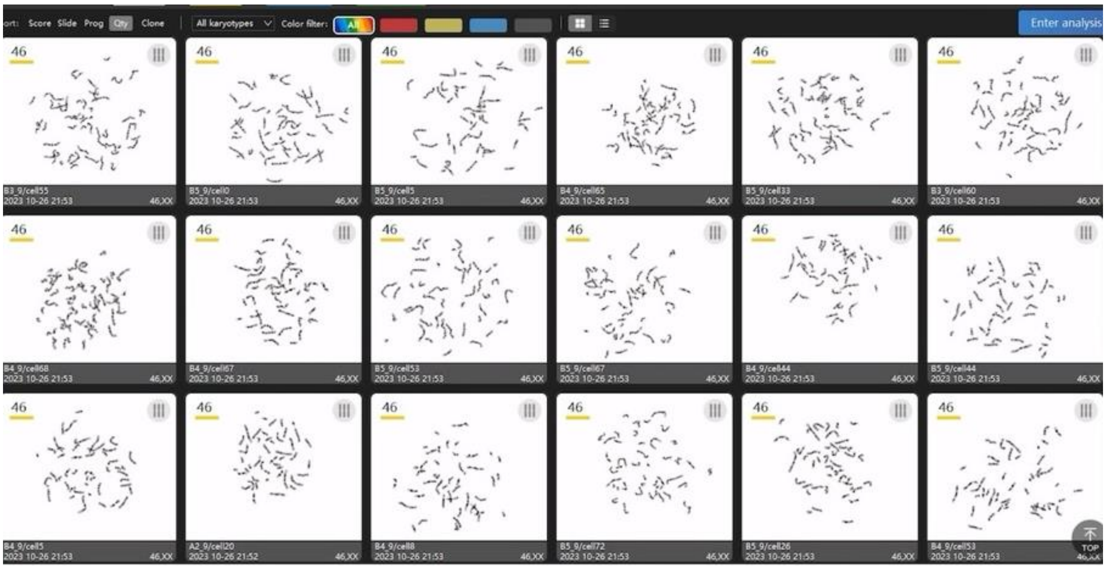

Supplemental References

Qin Y, Wen J, Zheng H et al (2019) Varifocal-Net: A Chromosome Classification Approach Using Deep. Convolutional Networks. IEEE Trans Med Imaging 38:2569–2581. https://doi.org/10.1109/TMI.2019.2905841

Hook EB (1977) Exclusion of chromosomal mosaicism: tables of 90%, 95% and 99% confidence limits and comments on use. Am J Hum Genet 29:94–97

Li J, Fu F, Wei R et al (2024) Chromosomal Structural Abnormality Diagnosis by Homologous. Similarity. In: Proceedings of the 30th ACM SIGKDD Conference on Knowledge Discovery and Data Mining pp 5317–5328. https://doi.org/10.1145/3637528.3671642
